# Protective effects of fecal microbiota transplantation against ischemic stroke and other neurological disorders: an update

**DOI:** 10.3389/fimmu.2024.1324018

**Published:** 2024-02-21

**Authors:** Tousif Ahmed Hediyal, C. Vichitra, Nikhilesh Anand, Mahendran Bhaskaran, Saeefh M. Essa, Pravir Kumar, M. Walid Qoronfleh, Mohammed Akbar, Ruchika Kaul-Ghanekar, Arehally M. Mahalakshmi, Jian Yang, Byoung-Joon Song, Tanya M. Monaghan, Meena Kishore Sakharkar, Saravana Babu Chidambaram

**Affiliations:** ^1^ Department of Pharmacology, JSS College of Pharmacy, JSS Academy of Higher Education & Research, Mysuru, KA, India; ^2^ Centre for Experimental Pharmacology and Toxicology, JSS Academy of Higher Education & Research, Mysuru, KA, India; ^3^ Department of Pharmacology, American University of Antigua, College of Medicine, Saint John’s, Antigua and Barbuda; ^4^ College of Pharmacy and Pharmaceutical Sciences, Frederic and Mary Wolf Centre University of Toledo, Health Science, Toledo, OH, United States; ^5^ Department of Computer Science, Northwest High School, Bethesda, MD, United States; ^6^ Molecular Neuroscience and Functional Genomics Laboratory, Department of Biotechnology, Delhi Technological University (Formerly DCE), Delhi, India; ^7^ Q3CG Research Institute (QRI), Research and Policy Division, Ypsilanti, MI, United States; ^8^ Division of Neuroscience and Behavior, National Institute on Alcohol Abuse and Alcoholism, National Institutes of Health, Bethesda, MD, United States; ^9^ Symbiosis Centre for Research and Innovation (SCRI), Cancer Research Lab, Symbiosis School of Biological Sciences (SSBS), Symbiosis International University (SIU), Pune, Maharashtra, India; ^10^ Drug Discovery and Development Research Group, College of Pharmacy and Nutrition, University of Saskatchewan, Saskatoon, SK, Canada; ^11^ Section of Molecular Pharmacology and Toxicology, Laboratory of Membrane Biochemistry and Bio-physics, National Institute on Alcohol Abuse and Alcoholism, National Institutes of Health, Rockville, MD, United States; ^12^ National Institute for Health Research Nottingham Biomedical Research Centre, University of Nottingham, Nottingham, United Kingdom; ^13^ Nottingham Digestive Diseases Centre, School of Medicine, University of Nottingham, Nottingham, United Kingdom

**Keywords:** gut microbiota, gut-brain axis, immune cells, neuroimmune axis, neuroinflammation, fecal microbiota transplantation, ischemic stroke, neurological disorders

## Abstract

The bidirectional communication between the gut and brain or gut-brain axis is regulated by several gut microbes and microbial derived metabolites, such as short-chain fatty acids, trimethylamine N-oxide, and lipopolysaccharides. The Gut microbiota (GM) produce neuroactives, specifically neurotransmitters that modulates local and central neuronal brain functions. An imbalance between intestinal commensals and pathobionts leads to a disruption in the gut microbiota or dysbiosis, which affects intestinal barrier integrity and gut-immune and neuroimmune systems. Currently, fecal microbiota transplantation (FMT) is recommended for the treatment of recurrent *Clostridioides difficile* infection. FMT elicits its action by ameliorating inflammatory responses through the restoration of microbial composition and functionality. Thus, FMT may be a potential therapeutic option in suppressing neuroinflammation in post-stroke conditions and other neurological disorders involving the neuroimmune axis. Specifically, FMT protects against ischemic injury by decreasing IL-17, IFN-γ, Bax, and increasing Bcl-2 expression. Interestingly, FMT improves cognitive function by lowering amyloid-β accumulation and upregulating synaptic marker (PSD-95, synapsin-1) expression in Alzheimer’s disease. In Parkinson’s disease, FMT was shown to inhibit the expression of TLR4 and NF-κB. In this review article, we have summarized the potential sources and methods of administration of FMT and its impact on neuroimmune and cognitive functions. We also provide a comprehensive update on the beneficial effects of FMT in various neurological disorders by undertaking a detailed interrogation of the preclinical and clinical published literature.

## 1 Introduction

The Gut-brain axis (GBA), also known as the microbiota-gut-brain axis, refers to the complex bidirectional connections between gut and brain occurring through the central, autonomic, and enteric nerves systems, endocrine system, and innate and acquired immune systems (1, 2). The gut microbiota (GM) refers to the collection of microorganisms such as bacteria, viruses, protozoa, archaea, and fungi, which are present in the highest density in the colon. The collective genome of the GM in the gastrointestinal tract (GIT) is called the gut microbiome (3). An imbalance between intestinal commensal and pathobionts leads to a disruption in the gut microbiota or gut dysbiosis (GD), which in turn affects gut-immune and neuroimmune systems. The crosstalk between the gut and brain suggests that the GM plays an important role in regulating the metabolism, immune system, and vascular system of the host (4).

### 1.1 Gut microbiota in host homeostasis

The human GM comprises 10–100 trillion microbes constituting a diverse population belonging to both commensal and opportunistic microbes (5). There are more than 100 bacterial phyla; the major two phyla are *Firmicutes* and *Bacteroidetes*, and minor phyla are *Actinobacteria, Proteobacteria, and Verrucomicrobia* (6). A compositional study on the gut microbiome by Hollister et al. revealed that sex, race/ethnicity, environmental factors, and age contribute to variations in the human GM composition (7). The GM and its metabolites mediate the wellness of the body by regulating immunological, metabolic, and neuroendocrinological functions. The functions of the GM and metabolites are diverse, ranging from supporting the absorption of nutrients and minerals, the synthesis of enzymes, vitamins, and amino acids, and the production of bacterial-derived metabolites such as short-chain fatty acids (SCFAs) (acetate, propionate, valeric acid, and butyrate), phenylacetylglutamine, and trimethylamine N-oxide (TMAO); all of which maintain host homeostasis (8). The GM therefore acts as a defense barrier by mediating colonization resistance (preventing pathogenic colonization) through competing for attachment sites and nutrients, and by secretion and production of antimicrobials (9). Bacterial-derived metabolites, particularly SCFAs, help to modulate the maturation and functions of microglia in the brain, implicating its regulatory potential in modifying neuro-inflammatory responses and thus play a role in neurodegenerative diseases (NDDs) (10). The GM also plays an important role in immunomodulation by altering B-cell activation to produce IgA and Group 3 innate lymphoid cells (immune cells found in barrier sites), gut-associated lymphoid tissues, macrophages, dendritic cells in the lamina propria, and effector and regulatory T cells (11). For example, GD is associated with numerous diseases, including Inflammatory bowel disease (IBD) (12), diabetes (13), Parkinson’s disease (PD) (14), Alzheimer’s disease (AD) (15), autism (16), multiple sclerosis (17), and stroke (18). These reports indicate a strong involvement of GD in various pathological conditions, particularly altering the inflammatory milieu. GD impacts neurological disorders mainly through altering immune responses. Similarly, use of FMT is shown to improve immune dysregulation in *Clostridioides difficile* infection (CDI) and other inflammatory diseases (19, 20). Herein, we comprehensively demonstrate the mechanistic impact of FMT in various neurological disorder with a central focus on inflammation.

### 1.2 Gut microbiota in major central nervous diseases

Dysregulation of the gut-brain axis has been implicated in various neurological disorders including Alzheimer’s disease (AD), autism spectrum disorder (ASD), Parkinson’s disease (PD), and stroke. These neurological conditions have been shown to cause changes in the bidirectional link, resulting in the emergence of brain-gut disorders including irritable bowel syndrome (IBS) (21). AD etiology has also been strongly linked to GD and microbial-derived metabolites that are directly linked with phosphorylated tau/Aβ42 accumulation in AD (22). Interestingly, *Helicobacter pylori* infection has been linked to AD pathology. It causes abnormal hyperphosphorylation of tau protein as well as the release of inflammatory mediators and β-amyloids (23). GD has increased recognition in PD wherein the initiation of α-synucleinopathy is produced in the enteric nervous system (ENS) during the early stages of the disease (24). PD patients experience a wide range of clinical symptoms including difficulties in swallowing, drooling, delayed gastric emptying, small intestinal bacterial overgrowth, and constipation (25). Furthermore, gut microbes *belonging to Prevotellaceae, Christensenella, Akkermansia, and Lactobacillus* were previously demonstrated to be as associated with PD (26, 27). On the other hand, several studies have reported the GM composition differences between ASD and healthy children, where studies have reported a reduced abundance of beneficial bacterium *Bifidobacterium*, and an increased abundance of pathobionts such as taxa belonging to Clostridium cluster XVIII and *Escherichia/Shigella* (28, 29). These reports indicate that the GM regulates GBA functions and the pathophysiology of neurological disorders.

### 1.3 Gut microbiota and the neuroimmune axis

The GM exerts diverse beneficial effects on brain health, the neuroimmune system, and defense against pathogenic infections (30). A well-coordinated inflammatory response within the brain is known as a neuroinflammatory reaction, and it is mediated by a variety of cell populations, including astrocytes and microglia. Astrocytes, microglia, and peripherally derived immune cells are the primary producers of inflammatory mediators such as reactive oxygen species, cytokines, and chemokines (31). Strong experimental evidence suggests that the GM and immune-mediated inflammatory responses interact in a complex, dynamic, and bidirectional manner. Germ-free animals serve as an excellent tool for demonstrating the interaction between the GM on the innate and adaptive immune systems in health and disease states (32).

The innate immune response of the gut mucosa is directed against one or more pathogens and antigens (33). The GM in association with antigen-presenting cells (APCs) protects the body against infection. Also, to maintain immune tolerance to the normal gut flora, the dendritic cells (DCs) of Peyer’s patches (lymphoid nodules embedded in the gut wall) produce anti-inflammatory cytokines e.g., interleukin (IL)-10 and TGF-β (34). In the adaptive immune system, CD4+ T cells play an important role. The lamina propria of the intestine contains the majority of the intestinal CD4+ T lymphocytes. Naive CD4+ T cells can develop into one of four main subtypes after being stimulated: T helper 1 (Th1), Th2, Th17, or regulatory T cell (Treg) (35). However, in GD, the adaptive immune system activates CD4+ T cells which further interact with intestinal immune cells such as Th1, Th2, Th17, and Tregs, which lead to neuroinflammation and neuronal death (36).

**Figure 1:**
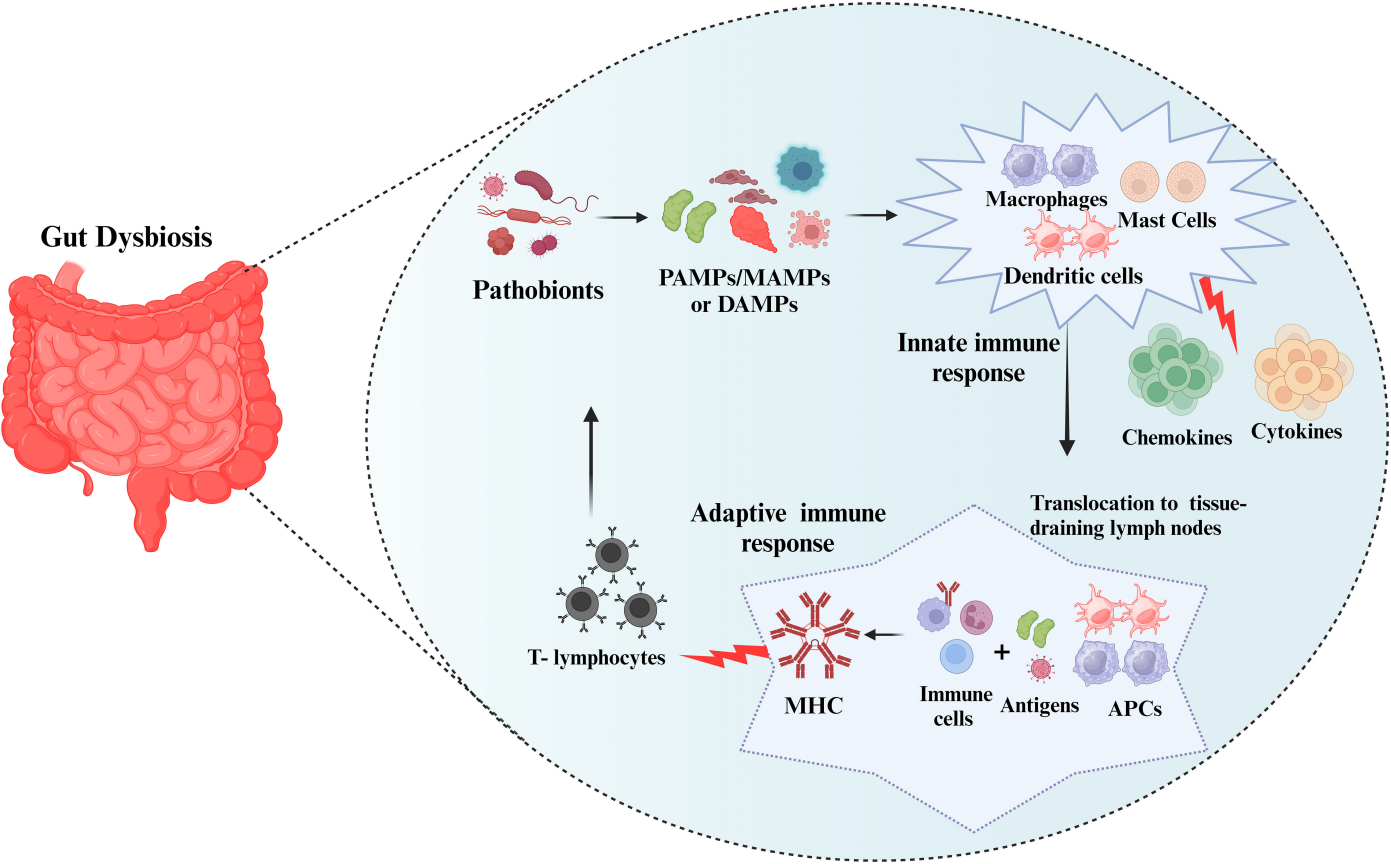
Immune response to the pathobionts. The pathobionts trigger inflammatory reactions via pathogen-associated molecular patterns/microbe-associated molecular patterns (PAMPs/MAMPs), or danger-associated molecular patterns (DAMPs). These stereotyped molecular patterns activate the innate immune response (mast cells and macrophages) and adaptive immune response resulting in the increased production inflammatory markers. Subsequently, antigen-presenting cells (APCs) translocate to tissue-draining lymph nodes and form major histocompatibility complex (MHC) and T-lymphocytes will fight against the pathobionts.

Pathogenic microbes/microbial components trigger inflammatory reactions via pattern recognition receptor (PRP) families that include pathogen-associated molecular patterns/microbe-associated molecular patterns (PAMPs/MAMPs), or by specific molecular structures released by host cells called danger-associated molecular patterns (DAMPs) (dead or dying cells, cancer cells, toxic, or allergenic structures (37). These stereotyped molecular patterns are recognized by tissue-resident immune cells (mast cells and macrophages) and as a result, they elicit an innate immune response resulting in the increased production of cytokines and chemokines and also induce complement activation (38). Localized dendritic cells and macrophages serve as antigen-presenting cells (APCs). To initiate an adaptive immune response, APCs translocate to tissue-draining lymph nodes where they expose local immune cells to foreign antigens via molecules of the major histocompatibility complex (MHC). A persistent inflammatory response results in the recruitment of circulating leukocytes, particularly T-lymphocytes, which enter the tissue and act as effectors of cellular adaptive immunity (**Figure 1**).

## 2 Evidence of gut dysbiosis in neurological disorders

Neurological disorders are closely associated with GD. The factors affecting GD are reduced abundance of beneficial bacteria, excessive proliferation of pathobionts, and a reduction in the overall microbial diversity in the gut (39). Numerous observational and animal studies suggest that the GM plays a vital role in the neuropathogenesis of CNS diseases via alteration in the GBA (40). GD leads to the production of metabolites and cytokines, initiating inflammation, influencing the BBB and brain volume, and potentially acting as pseudo neurotransmitters. These effects contribute to the disruption of brain physiology and neuronal function in various neurological disorders, including those characterized by demyelination or neuropsychiatric manifestations (41–43).

### 2.1 Cerebral stroke

The American Heart Association/American Stroke Association defines stroke as a “disease that affects the arteries leading to and within the brain”. Stroke remains the second leading cause of death globally, with high morbidity, disability, and recurrence rates (44). The World Stroke Organization reported over 12.2 million new stroke cases each year and more than 101 million people that are currently alive have experienced a stroke (45). Globally, 9.6 million cases of ischemic stroke and 4.1 million cases of hemorrhagic stroke have been documented, with 90% of these cases being triggered by modifiable risk factors. In India, stroke is the fourth and fifth leading causes of death and disability, respectively. The occurrence of stroke ranges from 105 to 152/100,000 persons annually in India (46).

In mice following stroke, T lymphocytes migrate from the small intestinal lamina propria or intestinal Peyer’s patches to the brain and/or the leptomeninges. Immune cells such as Th1 cells, Th17 cells, monocytes, and toxic chemicals from the gut, and bacteria migrate to the infarct region, which inhibits the migration of anti-inflammatory Treg cells (47). Pathobionts stimulate dendritic cell migration to mesenteric lymph nodes, which promotes T cells to differentiate into Treg cells (48). Impaired Treg cell migration to the lamina propria increases γδT-cell differentiation. The migration of these pro-inflammatory T cells from the lamina propria to the meninges increases the inflammatory milieu and infarct size (49). Additionally, the cytokines released by activated microglia and the DAMPs produced as a result of brain damage stimulate the vagus nerve, which ultimately results in GD (50). The increased translocation of bacterial toxins, pathogenic bacteria, and toxic metabolites produced by these alterations in the GIT subsequently leak additional gut inflammatory and immune cells into the bloodstream, leading to stroke-damaged areas (51). As a result, the outcome of post-stroke treatment is negatively impacted by the inflammatory loop which is triggered by GD. Cheng et al. demonstrated raised plasma levels of LPS and pro-inflammatory cytokines in cynomolgus monkeys post-stroke state, which corresponded with the relative abundance of the phylum *Bacteroidetes* (52). LPS is an immunogenic endotoxin produced by the Gram-negative bacteria (*Yersinia pestis, Klebsiella pneumonia, Pseudomonas aeruginosa, Porphyromonas gingivalis, bifidobacterial species, Chlamydia trachomatis and Francisella tularensis*) that can either directly cause neuroinflammation or peripheral immune cells to migrate into the brain (50).

Ischemic stroke is caused by atherosclerotic plaque or emboli occluding blood vessels, which reduces blood flow and oxygen availability, causing apoptosis and neuronal death (53, 54). Hemorrhagic stroke occurs as a result of deep perforating vasculopathy associated with high blood pressure, amyloid angiopathy of cerebral arteries with microbleeds, and clinical hemorrhages typically affecting the basal ganglia, cerebellum, pons, or thalamus (55). Clinical risk factors for stroke are hypertension, physical inactivity, unhealthy diet, tobacco use, use of alcohol, atrial fibrillation, raised blood lipid levels, genetic disposition, obesity, stress, diabetes, and depression. Post-stroke GI complications such as dysphagia, constipation, fecal incontinence, and GI bleeding are more frequently reported and are characterized by severe inflammation, bowel obstruction, and gut dysbiosis. These complications fuel disease progression and slow down the neuronal recovery of the degenerating brain (56).

Some studies have reported that GM composition influences stroke prognosis (57, 58). The GBA regulates immunological functions in the post-stroke condition while altered GM composition and abundance affect post-stroke outcomes mainly via gut leakiness, endotoxemia, and the neuroimmune axis. These mechanisms allow the dissemination and translocation of resident microflora, cellular, and humoral factors, including metabolites, immune cells, and cytokines/chemokines from the gut to the brain (59).

In ischemic stroke (IS), local and systemic inflammatory responses are augmented (60). Within 24 hours, monocytes (innate response) penetrate the brain, reaching peak numbers 3-5 days after acute ischemic stroke (AIS) (61). Neutrophils play a controversial role in AIS. Neutrophils have been linked to several mechanisms, including the generation of reactive oxygen species and the release of metalloproteinases, but it has also been shown that N2 neutrophils are neuroprotective (62). Additionally, neurotoxic mechanisms (oxidative stress, neuroinflammation, apoptosis, genotoxic activity, and impaired signaling pathways) activate the release of pro-inflammatory cytokines including interleukin-21 from a cluster of differentiating CD4+ T cells within 24 h post-AIS. Th1 and Th17 subpopulations promote neuroinflammation, whereas Tregs act as neuroprotectors by reducing inflammation (63). Moreover, T cells release cytokines and chemokines into the bloodstream which impact the microbiota composition and lead to GD (64). Stroke is frequently reported to be associated with gut dysmotility, increased intestinal barrier permeability, and translocation of microorganisms and a spike in the concentration of metabolites, such as TMAO and lipopolysaccharide (LPS), to enter the bloodstream (65, 66). These alterations further promote the systemic inflammatory response and disease progression and result in a poor prognosis.

In a Japanese cohort study in ischemic stroke patients, the relative abundance of *Atopobium cluster and Lactobacillus ruminis* were elevated and the relative abundance of *Lactobacillus sakeiakei* subgroup decreased and these changes triggered IL-6 detection in the circulation. Furthermore, the decreased concentration of acetic acid was negatively correlated with the levels of glycated hemoglobin and low-density lipoprotein cholesterol. In contrast, elevated fecal valeric acid concentration was positively correlated with the level of high-sensitive C-reactive protein and white blood cell counts. The changes in metabolites associated with GD in patients with IS are correlated with host metabolism and inflammation (67). These observations suggest a strong pathological involvement of GD in the IS condition.

### 2.2 Alzheimer’s disease

As in AD, GD causes altered release of neurotransmitters, which affects the GBA, resulting in behavioral changes, increased anxiety, mood swings, sleep deprivation, and depression. GD is primarily characterized by an elevation in the *Firmicutes/Bacteroidetes* ratio, which has been linked to the accumulation of gut APP from the early stages of AD (68). Alterations in the composition of the GM in APP/PS1 mice were linked to elevated Aβ levels in the CNS as well as impaired memory performances (69). Similarly, in Tg2576 animals, GD, intestinal epithelial barrier dysfunction, and vascular Aβ accumulation in the intestine occur before the development of cerebral Aβ depositions. The occurrence of Aβ deposition is also noted in intestinal autopsies of AD patients (22). Nevertheless, the relationship between GD, intestinal Aβ deposition, and AD onset still needs to be elucidated.

### 2.3 Parkinson’s disease

GD has been postulated to act as a potential cause of PD. Clinical studies have revealed the incidence of GD in PD patients compared with healthy individuals, and both fecal and mucosal GM compositions have been described to change in PD patients (27, 70, 71). Likewise, in the mouse model of PD (Thy1-αSyn transgenic animals), GM exacerbates motor impairments, microglial activation, and α-Syn accumulation. However, in the absence of GM, the severity of motor impairments decreased but did not eliminate (72). In the rat PD model (wild-type Groningen rats), the treatment with dopamine agonists showed improvement in the GD with decreased abundance of pathobionts (*Lachnospiraceae and Prevotellaceae*) and increased abundance of beneficial gut flora (*Lactobacillus and Bifidobacterium*) (73, 74). Hence, the evidence of both clinical and preclinical studies has confirmed that GD plays a crucial role in PD pathology. However, more research is required to study the GM changes which occur in the post-PD phase.

### 2.4 Autism spectrum disorder

Researchers have reported many reasons for the association of GD with ASD symptoms development. For example, colonization of germ-free mice (GF) with fecal microbiota from ASD children exhibits ASD-like behavior. Mice colonized with microbiota from ASD subjects exhibited increased autistic behavior compared to normal mice (75). ASD patients were shown to have altered GM composition when compared to normal children (76). However, no specific GM species are causally linked to ASD (28, 77). However, in ASD, the data consistently reports alterations in the phyla *Bacteroidetes/Firmicutes* ratios (28, 78). In addition, a few studies have demonstrated an increased abundance of *Candida albicans*, which produce ammonia and other toxins that are likely to cause autism-related behaviors, in the intestines of ASD children compared to non-ASD children (79, 80). Similarly, *S. cerevisiae* stimulates the production of TNF-α and IL-6 by altering immune functions via activation of TLR ligands, and it is thought these immunological mechanisms may play a role in the onset of ASD (81).

## 3 Fecal microbiota transplantation

Currently, several potential approaches, such as the use of antibiotics as prophylactics, fecal microbiota transplantation (FMT), prebiotics, probiotics, and dietary interventions, have been shown to reverse post-stroke gut GD and improve quality of life (56). FMT is a microbiota-based therapeutic intervention and involves the transfer of fecal matter from a carefully selected donor into the GIT of a recipient to directly modify the recipient’s microbial composition and provide a health benefit (82). FMT is shown to be an effective therapy for GI and non-GI diseases, including neurological disorders such as stroke (83). In the United States, FMT is regulated as a biological agent by the Food and Drug Administration (FDA) with the source of FMT being largely supported by “stool banks” operated by clinical investigators or by OpenBiome. Since the FDA classifies FMT as an investigational new drug (IND), physicians and scientists are required to submit an IND application (84). However, at the European Union level, FMT still occupies a regulatory grey area where it falls to member states to decide how to regulate FMT. For instance, the UK regulates FMT as a medicinal product, where it is recommended for the treatment of recurrent *Clostridioides difficile* infection (CDI) under national guidelines (85). In Italy and Belgium, FMT is regulated as a tissue or cells under their respective national tissue and cell legislation (86). In Australia, all FMT products are regulated as biologicals with the level of regulation varying with the level of external governance and clinical oversight (87, 88). China has formed an FMT consensus expert group under the Committee of Gut Microbiota in Chinese Society of Gastroenterology since 2016 (89). Elsewhere, the regulations regarding FMT are still taking shape (90).

**Figure 2:**
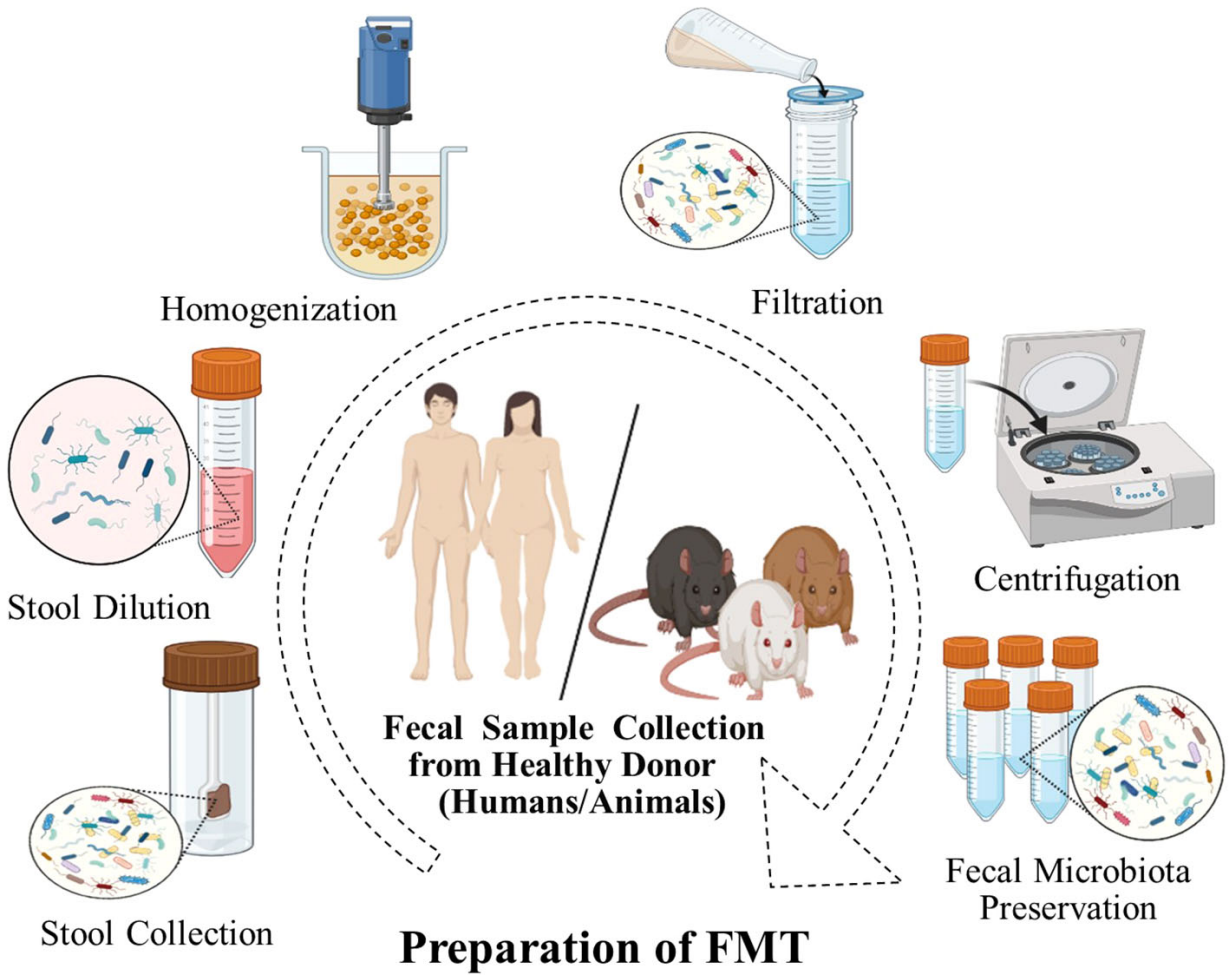
FMT preparation. A fecal sample is collected from donors, weighed, and dissolved in a fixed volume of sterile saline and homogenized. The mixture is filtered to remove large particulate matter, which may obstruct the endoscope channel. The filtrate is centrifuged and the supernatant is usually stored at -80°C until required for use.

The safety of FMT is an important factor in its implementation. Generally, FMT has been used to treat both GI and non-GI diseases and typically is associated with few or minimal side effects with strict donor screening (91–93) (**Table 1**). Most short-term risks are mild and known to be related to the delivery method. However, longer-term side effects have not been established (116). In addition, FMT has demonstrated variable degrees of efficacy in treating different GI problems and is the most effective therapy for the treatment of CDI. In the FMT process, stringent donor screening is necessary to avoid the transmission of infectious diseases. However, there is also a theoretical risk of FMT modulating susceptibility for developing conditions or diseases associated with the intestinal microbiota (117, 118). Donors must not have a family history of metabolic, autoimmune, and malignant conditions. Donors are also extensively screened for potential pathogens (85). The preparation of fecal matter involves the mixing of feces with water or normal saline and is followed by the removal of particulate matter through filtration, as shown in (**Figure 2**).

**Table 1:** FMT in GI and Non-GI Diseases: preclinical and clinical evidence.

Pre-Clinical Studies					
Disease types	Diseases	Species/Animal Model	FMT Route	Out Comes	References
GI Disorders	Ulcerative Colitis (UC)	Dextran sodium sulfate (DSS) induced UC in Balb/C mice	Enema	FMT reduced the severity of UC-related inflammation in mice, in which markedly decreased MPO activity, lowered TNF- and IL-1 levels, and enhanced IL-10 levels in colon tissue.	(94)
		GF BalB/c with DSS solution induced UC	Oral gavage	FMT significantly alters the metabolism of DSS-induced mice, and alleviate the symptoms of colitis	(95)
	*Clostridioides difficile* infection (CDI)	C57BL/6 mice induced with cefoperazone and 103 spores of *C. difficile* strain 630	Oral gavage	Conversely, mice who were not given FMT persistently colonized with high levels of *C. difficile*, and the GM in these mice persisted at low diversity	(96)
		C57BL/6J mice induced with antibiotics and 1.5 x 105 CFU	Enema	The efficacy of FMT products which have been frozen or lyophilized in treating *C. difficile* infection	(97)
Non-GI Disorders	Parkinson’s Disease	C57BL/6 mice induced PD with MPTP (i.p injection)	Oral gavage	FMT improved physical impairment in PD mice, lowered fecal SCFAs, reduced GM dysbiosis, boosted striatal DA and 5-HT content, reduced microglia and astrocyte activation in the substantia nigra, and decreased expression of TLR4/TNF-α signaling pathway components in the gut and brain.	(98)
		C57BL/6 mice induced PD with MPTP	Oral gavage	In substantia nigra, FMT suppressed the microglial and astrocytes activation. In addition, it downregulated the expression of GSK3β, IL-1β, inducible nitric oxide synthase and phosphorylated PTEN	(99)
	Alzheimer’s Disease	Male APPswe/PSEN1dE9 transgenic mice induced AD	Oral gavage	In transgenic mice, FMT therapy alleviated cognitive impairments and lowered amyloid- β (Aβ) brain deposition. It also boosted Synapsin I expression and decreased COX-2 and CD11b levels.	(100)
		5xFAD mice induced AD	Oral gavage	Following a 7-day FMT, significant alterations in amyloid plaque deposition and memory functions were seen in the 5xFAD mouse model of AD.	(101)
	Autism Spectrum Disorder	Sprague Dawley rats induced autism by propionic acid	Oral gavage	The amount of BDNF expression stabilized after FMT therapy restored the balance of fecal *Clostridium spp*.	(102)
		C57BL/6J male mice induced autism by p-cresol	Oral gavage	FMT of p-cresol treated mice to normal mice induces typical ASD behavior and increases p-cresol production. Nevertheless, FMT from healthy mice to p-cresol-treated animals was able to improve the social behavior, excitability of VTA dopamine neurons, and fecal p-cresol levels.	(103)
Clinical Studies					
Gastrointestinal Disorders	Ulcerative Colitis	Randomized clinical trial that included 73 pateints with mild to moderately active UC	Colonoscopy	In this preliminary study, anaerobically produced donor FMT was given for 1 week to patients with mild to moderate UC compared with autologous FMT. This resulted in a higher chance of remission at 8 weeks.	(104)
		A double-blind randomized controlled trial, 75 patients with active UC	Retention enema	FMT induces remission in a significantly increases the proportion of patients with active UC than placebo.	(105)
		A randomized control trial involving subjects with active UC	Colonoscopy for 1 day, then oral administration of frozen capsules	cFMT was related to long-lasting donor-induced changes in the microbial composition in fecal material. A correlation between changes in MAIT cell cytokine production and treatment response was found in cFMT recipients.	(106)
	CDI	Open-label single arm prospective, noncontrolled, pilot-study involving 7 patients with active UC	Oral capsules	In patients with active UC, 50 days of daily multidonor FMT capsules reduced F-calprotectin and temporarily improved symptoms and health-related life quality.	(107)
		Randomized, controlled, double-blind clinical trial, 46 subjects who had 3 or more recurrences of CDI	Colonoscopy	Colonoscopy-administered donor stool appeared to be safe and was more efficacious than autologous FMT averting further CDI episodes.	(108)
		Randomized clinical trial, involving 19 patients with CDI	Nasojejunal tube, superior endoscopy, or colonoscopy	Single FMT infusion was shown to have an efficacy of between 70 and 75 percent, and multiple infusions of the FMT enhanced this efficacy to between 85 and 90 percent.	(109)
Non- GI	PD	Case series involving 6 patients with PD	Colonoscopy	At 6 months, FMT was safe and improved PD’s non-motor and motor symptoms, including constipation.	(110)
		Prospective study included 11 PD patients with constipation	Nasoduodenal tube.	FMT significantly reduced both motor and non-motor PD symptomatology.	(111)
		Preliminary study including 15 patients with PD	Colonoscopy and nasal-jejunal tube	In comparison to nasointestinal FMT, the colonic FMT group demonstrated a considerable improvement and prolonged maintenance of efficacy. Also, it can safely decrease both the motor and non-motor symptoms experienced by PD patients.	(112)
	AD	A case report, 82-year-old man	Infusion	MMSE score, memory, cognition, mood, and social skills all improved after FMT.	(113)
	ASD	Open-label clinical trial involving 40 children with ASD	Capsules	FMT significantly altered neurotransmitters in the serum and improved the GI and behavioral indications of ASD patients.	(114)
		Follow up study, 18 patients diagnosed with ASD	Oral and rectal	FMT significantly improved GI and behavioral symptoms and the symptoms of autism improved significantly.	(115)

### 3.1 FMT delivery methods

**Figure 3:**
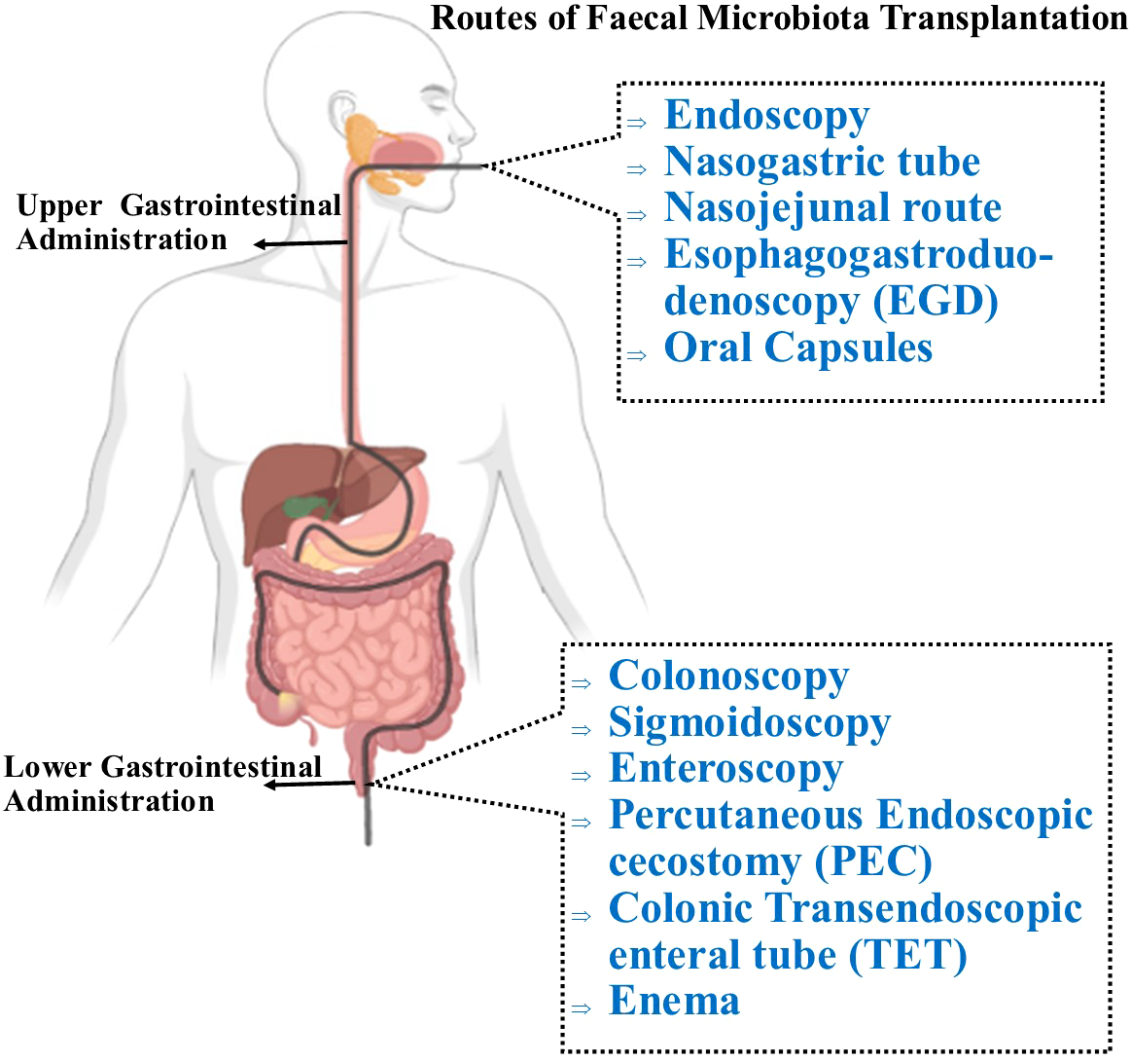
Routes of administration for FMT. Through the upper GI route, FMT can be administered via nasogastric tube which is placed through the nose and reaches the stomach or the jejunum (nasojunal tube) to deliver the fecal transplant. The most currently preferred delivery technique involves oral administration of encapsulated FMT, which is both safe and efficient, and is well-tolerated by patients. To deliver the processed fecal matter, the supply tube is inserted into the mouth and passed through the pharynx, oesophagus, stomach, and duodenum (EGD). In the lower GIT, FMT is administered with a colonoscopy, in which the physician inserts a colonoscope into the colon to administer the liquefied donor’s stool. Similar to a colonoscopy, a sigmoidoscopy primarily examines and delivers fecal materials to the lower third of the colon. A percutaneous endoscopic cecostomy (PEC) refers to when the C-tube is inserted under endoscopic or imaging guidance to the cecum. FMT can be delivered into the small intestine with esophagogastroduodenoscopy and using a longer conventional endoscope, a double-balloon endoscope (double-balloon enteroscopy) which may be passed through mouth or anus. During endoscopy, the colonic transendoscopic enteral (TET) tube is introduced into the ileocecal junction and connected to the cecum using titanium clips. The first technique to be used was the traditional enema, also referred to as a clyster, which involves injecting the stool preparation into the lower colon via the rectum.

FMT aims to treat a disease by restoring phylogenetic diversity and microbiota composition and function. Donor feces can be administered via the upper or lower GIT through different delivery routes (**Figure 3**) (119). In the upper GIT, FMT administration via capsule is relatively quick, convenient, inexpensive, and technically simple (120). The FMT procedure via the lower GIT necessitates bowel cleansing, followed by recolonizing the entire colon with favorable bacteria. This delivery route avoids the likelihood of aspiration and vomiting but requires a colonoscopy which is an invasive procedure (121). FMT enemas are easy to administer and are inexpensive. However, some patients have reported an aversion to handling stool, which obviously might interfere with the acceptability of fecal enemas (122). Although in the US, the FDA has reported six incidents of serious infections following FMT therapy (123), FMT is considered a safe, effective, and generally well-tolerated therapy with virtually no interim adverse effects if performed correctly. However, the evidence available on long-term safety is limited. Thus, it is crucial to establish clinical protocols that allow clinicians to operate with the highest degree of quality and safety assurance with the fewest possible risks associated with the process.

### 3.2 Role of FMT on neuroimmune functions

**Figure 4:**
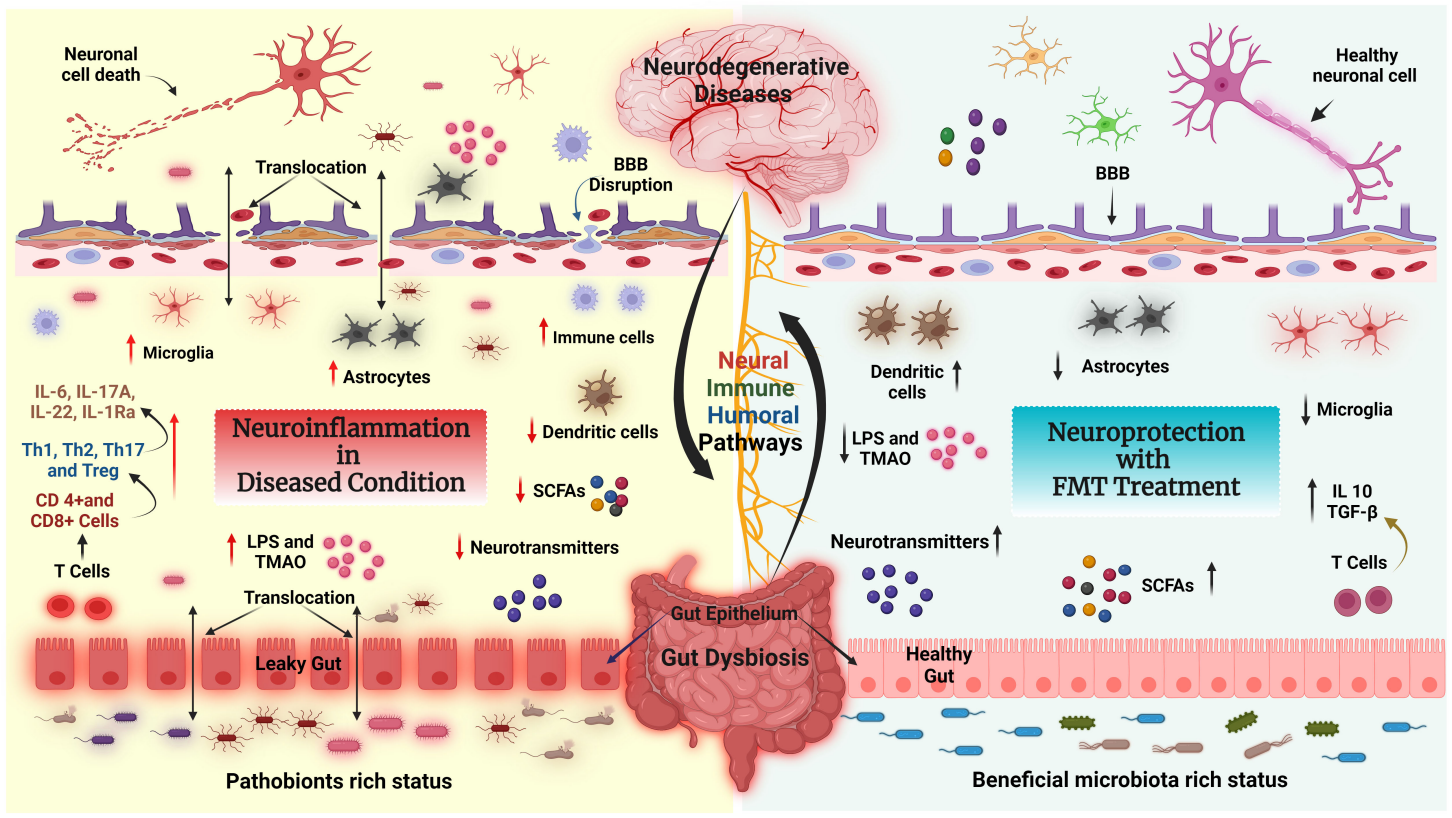
FMT modulates the neuroimmune axis. Effect of FMT on the immune system and metabolites in the context of neurological disease and gut dysbiosis (GD) state via the GBA. Left side: Pathobionts induce an inflammatory state during the GD condition. In GD, the adaptive immune system activates CD4+ T cells which further interact with intestinal immune cells [T helper 1 (Th1), Th2, Th17, or regulatory T cell (Treg)] and produce inflammatory cytokines such as IL-6, IL17A, IL22, IL-1Ra, LPS and TMAO, which leads to neuroinflammation and neural cell death. In addition, GD reduces the levels of fecal SCFAs, causing BBB disruption, activating astrocytes and microglia, and inducing neuroinflammation followed by neuronal cell death. Right side: The recolonized GM (beneficial GM) by FMT promotes the production of neurotransmitters, SCFAs, and regulatory T cells through interactions with intestinal immune cells (dendritic cells). Furthermore, FMT restores gut barrier integrity, decreases the production and translocation of TMAO and LPS to the periphery, reduces BBB disruption and activation of brain immune cells, and improves brain functions.

FMT increases intestinal microbial diversity and restores a healthy immune system (124). The potential therapeutic effects of FMT are correlated with decreased GD-induced neuroinflammation (**Figure 4**). For example, FMT therapy downregulates NLRP3 and IL-1 expression levels, which inhibit the NLRP3 inflammasome’s activation and prevents the appearance of pathogenic Th17 cells in a rat model of chronic cerebral hypoperfusion. In the intestinal mucosa, activation (phosphorylation) of Stat3 serves as a master regulator of Th17 cell development by upregulating the production of IL-6, IL-17A, IL-22, and RAR-related orphan receptor gamma (RORγt) (125). FMT treatment reduces the activation of microglial cells and inflammatory gene expression in a mouse model of traumatic brain injury (TBI) (126). Another study found that FMT reduced the proliferative capacity of colonic mucosal T cells in colitic mice compared with animals who did not receive FMT. The frequencies of CD8+ T and CD4+ T cells, which express the cytotoxicity-related molecule CD107a, were also decreased in FMT-treated animals. Indeed, colonic T cells from FMT animals treated showed a decreased pro-inflammatory phenotype (127).

In a clinical study, Jacob et al. performed a single FMT delivery by colonoscopy for active UC patients using a 2-donor fecal microbiota preparation. Post-FMT, mucosal CD4+ T-cell analysis showed a decrease in both Th1 and Treg cells (128). Crothers et al. reported that in a single-center prospective study, post-oral FMT treatment reduced the frequencies of Treg and mucosal-associated invariant T (MAIT) cell populations in the peripheral circulation of ulcerative colitis (UC) subjects (106). Wang et al. carried out a prospective study in UC patients, in which FMT administration significantly reduced the serum levels of IL-1Ra, IL-6, interferon-inducible protein (IP)-10, and epithelial neutrophil-activating peptide (ENA)-78. In addition, granulocyte-colony stimulating factor (G-CSF), vascular cell adhesion molecule (VCAM)-1, and mucosae-associated epithelial chemokine (MEC) levels were significantly decreased in sera from UC patients (129).

## 4 Evidence on the benefits of FMT in neurological disorders

### 4.1 FMT in stroke: preclinical evidence and outcomes

**Figure 5:**
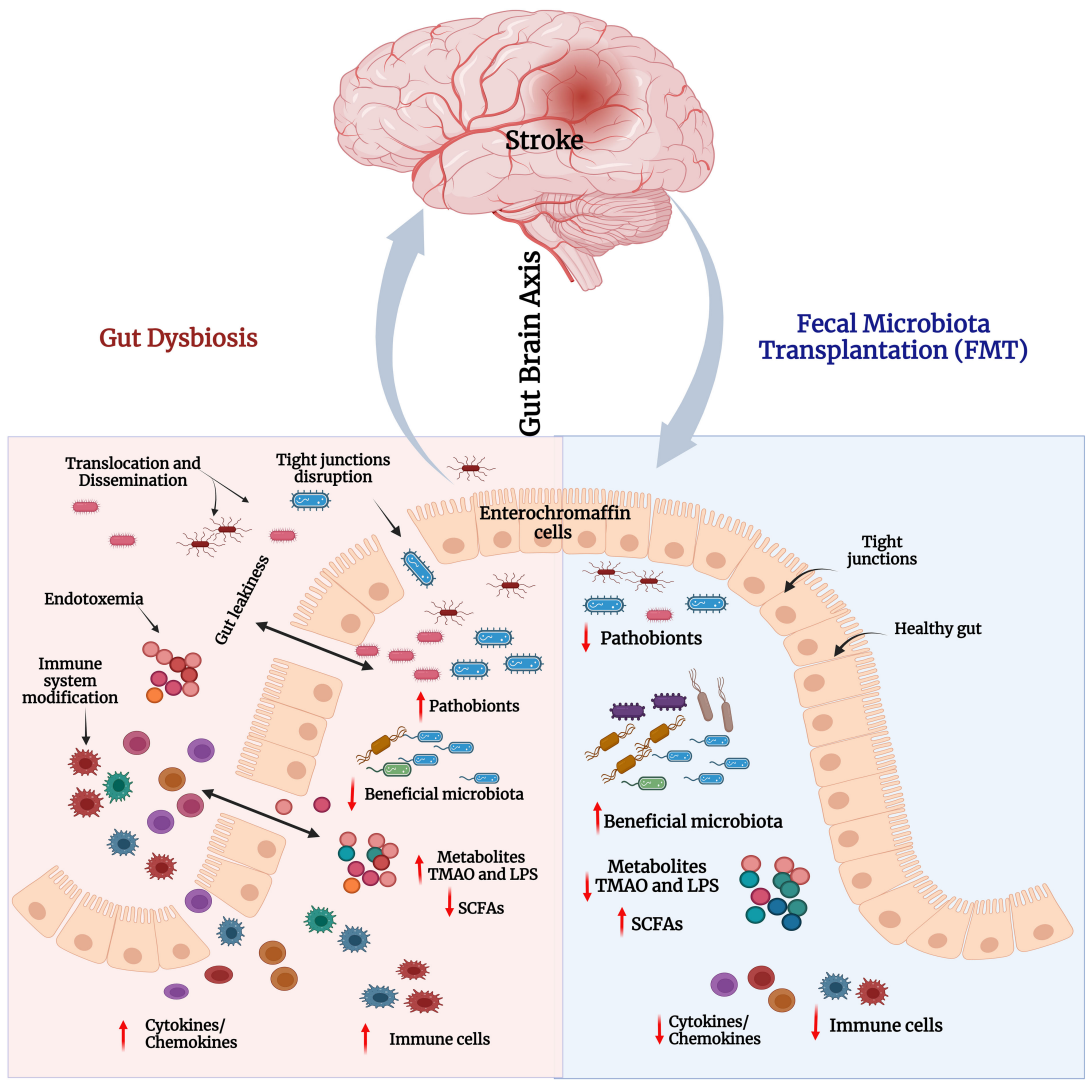
Gut dysbiosis and impact of FMT in stroke. Left panel – Stroke-associated gut dysbiosis leads to increased pathobionts and decreased beneficial bacteria in the gut. This leads to decreased levels of short-chain fatty acids (SCFAs), and elevated levels of LPS and TMAO. This triggers immune cells and the secretion of proinflammatory cytokines/chemokines. Increased cytokines and inflammation disrupt the tight junction integrity that induces gut leakiness. Consequently, excessive LPS, TMAO, cytokines, and immune cells pass through loose junctions into the systemic circulation to cause endotoxemia and systemic inflammatory reactions. Right panel - FMT reduces pathobionts and elevates abundance of beneficial bacteria i.e., restores gut microbial homeostasis and gut barrier integrity. Consequently, neuroinflammation and cognitive functions in stroke models would be improved.

In recent years, many animal experiments have been performed to define the relationship between GD associated with metabolic, neuropsychiatric, and sleep disorders, as well as intestinal and cardiovascular diseases (130, 131). Emerging evidence from animal studies has shown that manipulating the GM via FMT as a potential therapeutic approach for the treatment of stroke and post-stroke complications. A recent systematic review has summarized the preclinical animal evidence for the beneficial effects of FMT on stroke management (83). Inflammatory processes and altered metabolites have a detrimental impact on the GM in post-stroke conditions (132). In one study, post-stroke recolonization in germ-free mice resulted in increased levels of pro-inflammatory cytokines such as IFN-γ and IL-17, T-cell migration from the intestine to the post-stroke brain, and an aggravation of the lesion volume and functional impairments. In a separate study, FMT was associated with the reversal of GD and stroke outcomes with neuroprotective action (64). FMT in middle cerebral artery occlusion (MCAO) animal models fed a Western-style high-fat diet was found to inhibit apoptosis by lowering the level of Bax (Bcl-2-associated X protein) and cleaved caspase-3 and increasing Bcl-2, thereby attenuating the cerebral ischemic injury possibly via reduction in oxidative stress and brain apoptosis (133). In MCAO aged mice, FMT by gavaging stool from young mice was shown to improve post-stroke recovery and survival, while the converse was observed when FMT was administered from aged mice. In this latter group, FMT worsened post-stroke mortality indicating the role of age in affecting the gut microenvironment (134). Similarly, a gender-derived GM transplantation study revealed the dominance of microbial communities from female mice in increasing the survival rate, protection from brain damage and decreasing the systemic level of inflammatory cytokines in the post-stroke condition, suggesting sex as another critical factor influencing the gut microenvironment (135) (**Figure 5**, **Table 2**).

**Table 2:** Preclinical evidence of FMT in stroke outcomes.

Preclinical Evidences			
Study Design	FMT Procedure	Outcomes	References
C57BL/6 male aged and young mice MCAO Stroke model	Oral feeding	FMT from young mice had beneficial effects in MCAO aged mice (improved behavioral functions, cytokine levels, GM levels, infarct volume, SCFAs levels) when compared to aged animal’s FMT given to young MCAO mice.	(134)
Mice with MCAO-induced transient focal cerebral ischemia	Oral gavage	FMT improved the BBB, decreased the size of the infarct, improved gut barrier function, and decreased serum levels of LPS, LPS binding protein (LBP), and proinflammatory cytokines.	(136)
Sprague Dawley rats with MCAO induced ischemic stroke	Intragastric	FMT decreased intestinal permeability, T-CHO in serum, neurological impairment, and cerebral infarct volume.	(137)
C57 mice with MCAO induced ischemic stroke	Intragastric	FMT reduced the infarct area, improved behavioral test performance, decreased the level of inflammation, and enhanced the release of beneficial metabolites in female mice compared to male mice in a sex-dependent manner.	(135)
WT C56BL/6J and Rag1-/- mice and GF C56BL/6J and Rag1 -/- mice with MCAO induced cerebral ischemia	Oral gavage	FMT showed neuroprotective effect, reduced lesion size, and regulated T cells in peripheral immune system.	(64)
Mice with MCAO induced ischemic stroke	Oral gavage	Mice who received young donor FMT showed greater post-stroke behavioral improvement and less inflammation in the brain and intestine. Researchers also discovered that the young donor microbiota contained significantly more short-chain fatty acids (SCFAs).	(138)
Sprague Dawley rats with controlled cortical impact model induced TBI	Colonoscopy	After TBI, FMT reduced the TMA levels in the feces and TMAO in serum and ipsilateral and improved neurological deficits possibly via the TMA-TMAO-MsrA signaling pathway.	(139)

*Lactobacillus* species have been shown to decrease oxidative stress, neuronal apoptosis, and cerebral infarction volume via inhibition of TLR-4/NF-κB signaling, and prevented barrier dysfunction by repairing epithelial cells in the gut following cerebral ischemia reperfusion-injury (140). In antibiotic-treated C57BL/6 mice, transplantation of gut microbiota from db/db mice treated with sodium butyrate showed a reduction in cerebral infarct volume with better gut barrier and blood-brain barrier (BBB) function. These mice also exhibited an increased abundance of butyrate-producing bacteria such as *Ruminococcaceae, Ruminococcu, Lachnospiraceae, and Oscillospira* and showed reduced levels of serum LPS and inflammatory cytokines (IL-6, TNF-α, and IL-1β) in ischemic mice. Furthermore, attenuation of IS injury in the butyrate-treated mice correlated with reduced levels of systemic inflammatory markers such as intercellular adhesion molecule-1 (ICAM-1), vascular cell adhesion molecule-1 (VCAM-1), and matrix metalloproteinase-9 (MMP-9) in the brain. The latter may also be due to the protection of the BBB, marked by the prevention of cerebral capillary endothelial glycocalyx degradation, reflected by the lower levels of syndecan-1 and heparan sulfate in serum (136). FMT therapy improved GM-derived metabolites including SCFAs in the recipient group as compared to the saline group (137). SCFAs support the growth of beneficial bacterial populations such as *Lactobacillus, Butyricicoccus, and Meganonas*. Additionally, FMT also alleviated the level of serum total cholesterol levels, cerebral infarct volume, and intestinal permeability in an MCAO mouse model (137).

Based on these preclinical findings, GD exerts chronic inflammatory responses both peripherally and centrally that accelerate stroke prognosis. Certain bacterial phyla such as *Bacteroidetes, Firmicutes, and Actinobacteria* are altered in the post-stroke condition as depicted by increased F/B ratio (141), Therefore, targeting these bacterial phyla via FMT represents a promising adjuvant therapeutic and prophylactic strategy to protect the brain and gut against stroke-related complications.

### 4.2 FMT in stroke: clinical evidence and outcomes

The pathophysiology of stroke is strongly linked with gut inflammation and immune responses. This could be a key therapeutic target in post-stroke management. Studies reported that patients with stroke showed altered GM composition compared to healthy subjects, particularly in specific bacterial phyla as mentioned in (**Table 3**) (145). The GM-derived SCFAs alter brain functions directly or indirectly via immune, vagal, endocrine, and humoral pathways (146). Na Li et al. reported that in ischemic stroke patients, the fecal microbiota composition of SCFA-producing taxa *Odoribacter* and *Akkermansia* was decreased. Additionally, the genus *Christensenellaceae_R-7_group*, *norank_f_Ruminococcaceae*, and Enterobacter levels were correlated to stroke severity, whilst *Christensenellaceae_R-7_group* levels were positively correlated with IS outcomes (147).

**Table 3:** Preclinical and clinical studies showing alterations in GM composition after stroke.

Mode of the Study	Gut microbiota (Increased abondance)	References
Human cohort study (n= 140)	*Porphyromonadaceae* and *Enterobacteriaceae* and *Lactobacillaceae* and *Akkermansia*	(142)
Human cohort study (n=104) and MCAO study (mice)	*Butyricimonas, Parabacteroides, Rikenellaceae, Ruminococcaceae, Oscillospira, Bilophila, Enterobacteriaceae*	(143)
Mouse MCAO model	*Akkermansia muciniphila* and *clostridial species*	(144)
Human case-control study (n=435)	*Enterobacter, Oscillibacter, Megasphaera,* and *Desulfovibrio*	(145)

In acute ischemic stroke patients, there is an abundance of Enterobacteriaceae and decreased levels of *Fecalibacterium, Parabacteroides Clostridiaceae, and Lachnospira*. Animals receiving FMT from subjects with a high stroke dysbiosis index (SDI) showed a higher abundance of *Enterobacteriaceae* and lower levels of *Lachnospiraceae* which are associated with severe brain injury and poor stroke outcomes, in part displayed by a downward trend of T cells which act as neuroprotectants (143).

In stroke, thrombin causes direct vascular disruption, cellular dysfunction, oxidative stress, and inflammation. In addition, there is a direct correlation between thrombin activity in the affected brain hemisphere and the infarction volume (148). Yassene Mohammed et al. reported that the FMT from healthy donors suppressed the onset of thrombin generation in metabolic disorder patients (149). However, the effect of FMT on delaying the onset of thrombin generation in stroke patients is yet to be investigated.

Despite the different aetiologies of neurological disorders, neuronal damage is commonly associated with chronic activation of an innate immune response in the central nervous system (CNS). The GM composition differs in patients compared to healthy controls, indicating a pathophysiological role of the gut microbiome in CNS functions. However, GM restoration by FMT could be an effective treatment approach for several neurological disorders despite the available limited evidence (113).

### 4.3 Alzheimer’s disease

Alzheimer’s Disease (AD) is characterized by the accumulation of specific proteins inside or outside cells such as misfolded amyloid-β (Aβ) and tau hyperphosphorylation which forms neurofibrillary tangles. The GM plays a vital role in brain functions including myelinization, neurogenesis, and microglial activation which are closely related to behavioral, mood, and cognitive modulations (130). Mounting evidence indicates that AD is associated with abnormal GM (150). The abundance of *Desulfovibrionaceae* and *Helicobacteraceae* at the family level and *Helicobacter* and *Odoribacter* at the genus level were significantly increased in APPswe/PS1dE9 transgenic (Tg) mice compared with wild-type (WT) mice (69). In an APPswe/PS1dE9 Tg mouse model, FMT improved cognition as evidenced by increased synaptic markers (PSD-95, synapsin-1) and decreased Aβ accumulation and neuroinflammatory markers (COX-2, CD11b). FMT treatment enriched bacterial like *Proteobacteria, Verrucomicrobia*, at phylum levels and *Desulfovibrio and Akkermansia* at genus levels and decreased the abundance of *Bacteroidetes* at phylum levels *and Alloprevotella* at genus levels in the Tg animals (100). Furthermore, FMT restored the levels of *Acidobacteria* and *Bifidobacterium* which significantly delayed AD progression in 3xTg-AD mice (151). Dodiya et al. reported that FMT between age- and sex-matched AD male mice without antibiotics and antibiotic-treated AD male mice partially restored microglial morphology and recolonized intestinal bacteria. Post-FMT in antibiotic-treated male mice, anti-inflammatory cytokines such as IL-10 were expressed more frequently, while pro-inflammatory cytokines like IL-1β, IL-2, IL-3, IL-17A, LIX (CXC5), RANTES (CCL5) and a cluster of differentiation 30 (CD30) and CD40 were reduced (152). Elangovan et al. demonstrated the 5XFAD mouse model showed significantly improved novel object recognition and spatial memory and reduction in amyloid pathology after 7 days of treatment of FMT from wild-type donor mice (101). These data demonstrate that FMT is an effective treatment for AD-like disorders, but further studies are required.

### 4.4 Parkinson’s disease

PD is a chronic, progressive, neurodegenerative condition characterized by accumulation of the presynaptic neuronal protein α-synuclein (αSyn) and dopaminergic neuron loss (110). It is not only associated with motor and non-motor deficits but also affects GI function causing altered bowel movement patterns and abdominal bloating (153). Several studies have reported a relationship between Toll-like receptors (TRLs), particularly TRL4, GM, and αSyn pathology. TLR4 depletion inhibited upward regulation of AP-1 in dopaminergic neurons in the substantia nigra of PD mice, suggesting that TLR4 may play a potential role in PD (154). TLRs have also been demonstrated to activate several signaling pathways, including PI3K/AKT/GSK3 and NF-κB. Additionally, TLR4 activation stimulates microglial cells and α-syn, which in turn enhances the synthesis of TNF-α and nuclear translocation of NF-κB (155).

Studies have reported that patients with PD have an increased abundance of genera including *Akkermansia, Lactobacillus, Bifidobacterium, Enterobacteriaceae*, and decreased levels of *Blautia, Faecalibacterium,* and *Prevotella* compared to healthy controls (14, 156). Very recent reports also reveal that FMT treatment improved the motor and non-motor functions in PD patients and reduced the symptoms of constipation (110, 157). FMT treatment inhibited the expression of p-PI3K, p-AKT, TLR4, and NF-κB, as well as TNF-α, confirming a close link between the TLR4/PI3K/AKT/NF-κB signaling and gut microbial dysbiosis in PD (158).

### 4.5 Autism spectrum disorder

Autism spectrum disorder (ASD) are severe brain or neurodevelopmental disorder that is characterized by deficits in social communication with restricted, repetitive, and stereotyped behaviors that can vary in individuals along a continuum of severity. The pathophysiology of ASD is significantly influenced by the neuroimmune system’s involvement and dysregulation, which stimulate microglia, astrocytes, and the release of pro-inflammatory cytokines (159). Inga J´acome et al. reported that in patients (3 to 9-year-old children) with ASD, concentrations of pro-inflammatory cytokines such as IL-1β, IL-6, IL-17, IL-12p40, and TNF-α in plasma were high and correlated with disease severity and progression in comparison with healthy subjects (160). Additionally, children with autism have also shown signs of immune system activation, such as an aberrant CD4:CD8 T cell ratio, a high proportion of DR+ (activated) T cells, elevated neopterin levels in the urine, and increased cytokine production (161). The systemic and neuroinflammatory effects of GM are anticipated to have a direct impact on lymphoid cells and the adaptive immune response. In general, microbial components are known to directly interact with antigen-presenting cells, TLRs, differentiated B cells, T cells, and CD4+ T cells (162). The GM induces activation of Th17 and Th17 lymphocytes which increases systemic inflammation and promotes BBB disruption and CNS inflammation (163).

In recent years, emerging research reported the association between ASD and GM, which was also identified as a contributing component to the prognosis of ASD through the GBA (28, 164). Patients with ASD had higher abundance of *Bacteroides, Parabacteroides, Faecalibacterium, Clostridium,* and *Phascolarctobacterium*, according to a metanalysis report. On the other hand, control subjects have higher levels of *Coprococcus* and *Bifidobacterium* (164). In a clinical cohort study of ASD patients, Ning Li et al. found that the levels of the phyla *Verrucomicrobia*, and genera *Eubacterium Coprostanoligenes, Akkermansia, Ruminococcus, Corprococcus,* and *Christensenellaceae* were significantly altered and that of 5-HT transporter (SERT or 5-HTT) or 5-HT levels were elevated. FMT treatment through oral and rectal delivery led to significant improvement (gastrointestinal symptoms and autism-like behaviors) in ASD patients by reducing *Eubacterium* and *Coprostanoligenes*. In addition, FMT decreased the serum levels of neurotransmitters 5-HT and GABA (114). In a single case report, FMT by colposcopy and oral capsule was shown to improve gastrointestinal and ASD-related symptoms and decrease the Childhood Autism Rating Scale (CARS scores) in a patient with ASD (165).

FMT from healthy human GM significantly reduced anxiety-like repetitive behaviors and raised serum levels of chemokines, including GRO-1 (CXCL1), MRIP-1 (CCL3), MCP-3 (CCL7), Eotaxin (CCL11) and RANTES (CCL5) which are crucial for neurogenesis and synaptic transmission in the central nervous system, in ASD mice (166). In another study, FMT from naive wild-type mice significantly reduced levels of TNFα and Iba1 in the mouse brain, normalized *A. muciniphila* abundance to wild-type levels, and restored memory impairments and social disengagement in *Fmr1* knockout mice (167).

The GM affects numerous essential host functions, including the immune response and the nervous system. Both human and animal data do seem to indicate that FMT may improve ASD symptoms. However, as data on the safety profile of FMT and the long-term effects of this treatment in ASD is still limited, further research is needed.

## 5 Conclusion

In the current review, we have summarized the relationship between GD and the development and prognosis of various neurological diseases including AD, PD, ASD, and stroke. The GM may be considered a potential contributory factor in the pathogenesis of neurological disorders. Restoration of the GM by FMT may attenuate symptoms or progression of neurological disorders mainly via GM-mediated immunological and neural pathways. Neurodegenerative diseases have yet to attract effective disease-altering/modifying therapies, despite decades of intensive research. Current therapies typically try to relieve symptoms but impose severe adverse reactions that restrict usage. Thus, there is an essential need for microbiome-inspired therapeutic alternatives that enhance the quality of life or significantly alter the course of the disease. The information compiled in this review indicates that FMT may be a viable and promising therapeutic option for treating several GI and neurological diseases. Nevertheless, large double-blinded randomized clinical tests (RCTs) are desirable to further elucidate the effect of FMT in numerous disorders of the microbiota GBA.

There is a lack of molecular understanding, despite clinical and preclinical data supporting the use of FMT in neurological diseases. Most studies did not investigate the possible detrimental effects of FMT, and long-term adverse effects were seldom identified. The efficacy of FMT is influenced by several factors, including donors, antibiotic types, treatment approaches, and microbial composition. To further validate the safety and rule out any potential adverse effects, long-term follow-up and appropriate controls are required. In addition, there has been no uniformity in dose, FMT delivery route, stool filtering technique, frequency of administration, and diet in the studies. Also, there is currently no approved standard for choosing donors for specific disorders, thus more research in this line is necessary. Several studies have shown that FMT improves neurological disorders by improving GI symptoms, and host immune function. However, in future, robust taxonomic resolution methods may be employed for identifying the biomarkers for specific neurological disorders. Finally, further mechanistic studies are warranted to understand the direct neuroprotective effects of specific species or bacterial-derived metabolite(s) on the gut-brain axis using molecular techniques such as human organotypic cultures, synthetic communities and microbiome depleted germ free mouse models to determine causality.

## Author contributions

TH: Formal analysis, Methodology, Software, Writing – original draft, Investigation, Project administration. CV: Writing – original draft. NA: Writing – original draft. MB: Writing – original draft. SE: Investigation, Writing – original draft. PK: Writing – original draft. MW: Writing – original draft. MA: Writing – original draft. RK-G: Writing – original draft. AM: Writing – original draft. JY: Writing – original draft. BS: Data curation, Investigation, Supervision, Writing – review & editing. TM: Conceptualization, Data curation, Supervision, Writing – review & editing. MS: Conceptualization, Data curation, Supervision, Writing – review & editing. SC: Conceptualization, Data curation, Supervision, Writing – review & editing.
